# Normative data of the Spanish version of the Montreal Cognitive Assessment (MoCA) in older individuals from Peru

**DOI:** 10.1590/1980-5764-DN-2024-0261

**Published:** 2025-05-19

**Authors:** Lucia Bulgarelli, Emilia Gyr, Jose Villanueva, Koni Mejía, Claudia Mejía, Renato Paredes, Sheyla Blumen

**Affiliations:** 1 Independent researcher, Lima, Peru.; 2 Pontifical Catholic University of Peru, Lima, Peru.; 3 EDMECON Educación Médica Continua, Lima, Peru.

**Keywords:** Mental Status and Dementia Tests, Dementia, Cognitive Dysfunction, Aged, Neuropsychology, Pruebas de Estado Mental y Demencia, Demencia, Disfunción Cognitiva, Anciano, Neuropsicología

## Abstract

The Montreal Cognitive Assessment (MoCA) has proven to be an effective tool for detecting early dementia in older adults. However, in the Latin American region, there are few available norms of the Spanish version. The Peruvian population faces major healthcare barriers to early screening, so it is important to validate this instrument within this population.

**Objective:**

To standardise the Spanish version of the MoCA for the older population in Lima, addressing the critical need for culturally and demographically adapted cognitive evaluation tools in Peru.

**Methods:**

The test was administered to 338 ambulatory and homebound adults aged 60 to 80 (216 women) from three institutions: San Miguel District Municipality, San José Obrero Polyclinic in Barranco, and EDMECON in Surco. we computed regression-based norms adjusted for age and education.

**Results:**

Sex was not a predictor of the total scores of MoCA. Moreover, age (R2=0.12) and education (R2=0.24) significantly influenced cognitive performance, with education being the strongest predictor. A raw score above 18 indicates normal cognitive performance for the total sample.

**Conclusions:**

We provided normative data and cutoff scores for older Peruvians, supporting the clinical use of the MoCA in Peru and setting a benchmark for future test standardizations in the region.

## RESUMO.

La Evaluación Cognitiva de Montreal (MoCA) ha demostrado ser una herramienta eficaz para detectar demencia temprana en adultos mayores. Sin embargo, en la región latinoamericana hay pocas normas disponibles de la versión en español. La población peruana enfrenta importantes barreras de salud para la detección temprana, por lo que es importante validar este instrumento dentro de esta población.

**Objetivos:**

Este estudio tuvo como objetivo estandarizar la versión en español de la MoCA para la población mayor de Lima, abordando la necesidad crítica de herramientas de evaluación cognitiva adaptadas cultural y demográficamente en Perú.

**Métodos:**

La prueba se administró a 338 adultos ambulatorios y confinados en sus hogares de 60 a 80 años (216 mujeres) de tres instituciones: Municipalidad Distrital de San Miguel, Policlínico San José Obrero en Barranco y EDMECON en Surco. Calculamos normas basadas en regresión ajustadas por edad y educación.

**Resultados:**

El sexo no fue un predictor de los puntajes totales del MoCA. Además, la edad (R2=0.12) y la educación (R2=0.24) influyeron significativamente en el desempeño cognitivo, siendo la educación el predictor más fuerte. Un puntaje bruto superior a 18 indica un desempeño cognitivo normal para la muestra total.

**Conclusión:**

Proporcionamos datos normativos y puntajes de corte para peruanos adultos mayores, lo que respalda el uso clínico del MoCA en Perú y establece un punto de referencia para futuras estandarizaciones de pruebas en la región.

## INTRODUCTION

The prevalence of dementia among individuals aged 60 years and older is approximately 8.5%^1^ in Latin America, with projections indicating an increase from 7.8 million in 2013 to 27 million by 2050^2^. The Global Burden of Disease (GBD) study shows that neurodegenerative diseases in Peru, measured in Disability Adjusted Life Years (DALY), rose from the fifth to the third largest contributor to neurological diseases^3^. A recent study in Peru^4^ found a 6.85% prevalence of dementia in 1,532 individuals over 65 in Lima, with Alzheimer's disease (AD) as the predominant diagnosis.

Dementia remains an incurable condition, with existing treatments primarily aimed at decelerating its progression^5^, thereby underscoring the significance of early diagnosis for effective rehabilitation. Early diagnosis provides numerous benefits to the patient:

- enhanced and more effective adherence to pharmacological and rehabilitative treatments;
- the opportunity to engage in experimental treatments;
- the possibility to plan one's life in accordance with the progression of the disease^6^.

Unfortunately, diagnosing dementia in Latin America faces critical challenges, as tests are rarely performed in primary care, leading to a high number of unnoticed and misdiagnosed cases^7^.

A promising alternative for the detection of early cognitive impairment in this region is the Montreal Cognitive Assessment (MoCA). The MoCA is a brief, reliable and valid test for neurocognitive evaluation, with a high degree of sensitivity and specificity for MCI and dementia^8,9^. The MoCA has demonstrated being superior to the Mini-Mental State Examination (MMSE) due to its design focused on identifying early stages of dementia^10^. Currently, the MoCA is available in multiple translated versions and over 86 cultural adapted versions unevenly distributed around the globe^11^, and has a known record of success in culture-sensitive adaptations^12^.

Prior studies have validated and standardized the MoCA within Brazilian^13-15^, Chilean^16^, and Argentinian^17^ cohorts. These studies indicate that the initial cutoff score of the test appears insufficient for accurately assessing populations with heterogeneous sociodemographic characteristics, particularly individuals with fewer years of formal education^13-15^, demonstrating a pronounced educational bias inherent in the test^17^. This could lead to a high risk of false positives and low accuracy in detecting dementia^13,17^. European normative studies have similarly identified limitations related to the diagnostic accuracy of the MoCA cutoff scores^18-20^, and advocate for thorough adjustments considering sociodemographic factors to mitigate these challenges. Advancing the validation of short cognitive assessments for the Peruvian population is critical given the diversity of cultural, demographic, and linguistic factors that may potentially affect test scores^21^. Peru lacks comprehensive neuropsychological test validation projects akin to those executed in other Spanish-speaking nations, such as the NEURONORMA initiatives in Spain^22^ and Colombia^23^. To date, a few short cognitive tests have been validated in the context of Lima-Peru, including the Clock Drawing Test (CDT)^24^; the Addenbrooke's Cognitive Examination (ACE)^25^; the INECO frontal screening test (IFS)^26^; and the memory impairment test (M@T)^27^. Nevertheless, the MoCA test has not been validated in the Peruvian context, and we still lack norms to interpret the scores in these population, leading to practices that are not in compliance with international early diagnostic standards^28^.

The objective of this study was to standardise the Spanish version of the MoCA for the older population in Lima-Peru, with an evaluation of the impact of influences exerted by age, gender, and educational attainment. Furthermore, normative data for the older population in Lima were established, considering variables such as age and educational levels.

## METHODS

### Study design

We conducted a transversal study including participants from three institutions within Metropolitan Lima: the EDMECON medical centre, the San José Obrero Polyclinic, and the San Miguel Municipality. Contact with participants was conducted within the context of a medical appointment at the three institutions. The study design and participants’ informed consent were approved by the Ethics Committee for Research in Social Sciences, Humanities and Arts of the Pontifical Catholic University of Peru, under approval number 145-2023-CEI-CCSSHHyAA. The MoCA was administered and scored by two physicians at EDMECON Medical Centre and two licensed psychologists at San José Obrero Polyclinic and the San Miguel Municipality, all of whom were trained in the administration of the test by a licensed clinical neuropsychologist. This study was granted permission to use the MoCA© for research purposes.

### Participants

The sample consisted of 338 adults over 60 years of age (122 male, 216 female), including both native Spanish speakers and those who spoke Spanish as a second language (with proficiency exceeding ten years). Participation in the study was voluntary and all participants gave their informed consent indicating their understanding of the objectives of the study and their willingness to participate. Sociodemographic data, including variables such as sex, age, date of birth, years of education, and medical diagnoses, were systematically collected. Age was divided into five ranges: 60–64, 65–69, 70–74, 75–79, and 80+ years after the research conducted by the National Institute of Informatics and Statistics (INEI) of Peru^29^. Similarly, years of education were divided into three levels: primary (0–6 years), secondary (7–11 years) and higher (12+ years), following the education levels reported by INEI^30^. A total of 17 participants, who were diagnosed with either neurodegenerative or sensory disorders by their respective healthcare institutions, were excluded from the sample.

### Measurement

We utilised the free Spanish MoCA version (from www.mocacognition.com), as used in other Latin American standardizations^16^. MoCA is a validated cognitive screening tool renowned for its efficacy in the early detection of dementia^9^. The test takes about 10 minutes and assesses eight cognitive domains: visuospatial and executive function, naming, memory, attention, language, abstraction, and orientation, with a maximum score of 30.

### Data analysis

We examined the potential effects of age, years of education, and sex in the raw scores of MoCA for the total sample. we evaluated sex differences in the total score and each of the cognitive domains of the MoCA using the Mann-whitney U tests. we used the Bonferroni correction of *p*-values to control for the family-wise error rate of multiple comparisons.

We explored associations of age and years of education with the total score and each of the cognitive domains of the MoCA using the Kendall correlation coefficient (τ_b_) and the determination coefficient (*r* ^2^). Here, we employed the Bonferroni correction to control for the family-wise error rate for each test. Potential differences in cognitive performance according to age group and educational level were analysed using the Kruskal-wallis H test and Bonferroni corrected post hoc Dunn tests.

Finally, we computed regression-based normed scores of the total MoCA scores adjusted for age and years of education following the procedure described in the NEURONORMA project^22^. First, raw scores were converted to percentile ranks (Pc). These ranks were rescaled to a truncated normal distribution (M=10, SD=3) bounded between 2 and 18 to generate scaled scores adjusted for age (*SS*_a_). This procedure was used for the entire sample and each age group.

Next, adjustments for years of education were modelled using a linear regression (Equation 1):


(1) $$SS_{\text{a}}=\text{α}+\text{β}*EducYears+\epsilon$$

The estimated regression coefficient (β) here was employed to calculate scaled scores adjusted for age and years of education (*SS*_ae_)^31^ (Equation 2):


(2) $$SS_{\text{ae}}=SS_{\text{a}}-\text{β}*(EducYears-11)$$

We selected a cut-off point of 11 years of education because it represents the period of basic education (primary and secondary) in Peru.

## RESULTS

**Figure 1:**
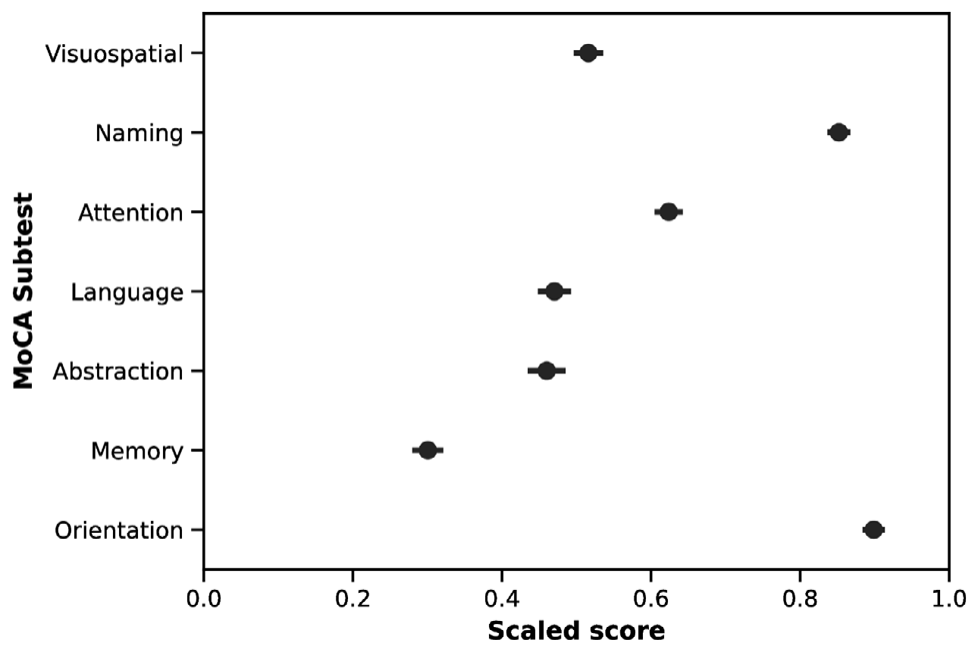
Scaled scores of Montreal Cognitive Assessment (MoCA) subtests.

The sociodemographic characteristics of the 338 participants in terms of age, education level, and sex are shown in Table 1. The mean cognitive performance of the total sample was 18.2±5.6 for the total MoCA score. The min-max scaled scores for each subtest are shown in Figure 1.

**Table 1:** Sociodemographic characteristics of the sample.

		Male (n=122)		Female (n =216)		Total (n=338)		*(p)*
		n	%	n	%	n	%	
Age group (years)	60–64	11	9	40	18.5	51	15	8.0 (0.08)
	65–69	26	21.3	44	20.3	70	20.7	
	70–74	24	19.6	49	22.6	73	21.5	
	75–79	22	18	35	16.2	57	16.8	
	>80	39	31.9	48	22.2	87	25.7	
Educational level (years)	Primary (0–6)	19	15.5	50	23.1	69	20.4	3.3 (0.18)
	Secondary (7–11)	51	41.8	90	41.6	141	41.7	
	Higher (>12)	52	42.6	76	35.1	128	37.8	

### Effects of sex, age and education

We did not find significant differences in the total scores of MoCA grouped by sex. However, we observed that men scored higher on identification (U=10,984.5, *p*=0.023) and attention (U=10,540.5, *p*=0.015) than women. No significant differences were found for other cognitive domains (Table 2).

**Table 2:** Montreal Cognitive Assessment (MoCA) scores grouped by sex.

Sex		MoCA Visosp/Exe	MoCA Identiﬁcation	MoCA Attention	MoCA Language	MoCA Abstraction	MoCA Delayed recall	MoCA Orientation	MoCA Total
Male (n=122)	Median	3	3	4	2	1	1	6	19
	IQR	2	1	2	1	2	3	0.75	6
Female (n=216)	Median	2	3	4	1	1	1	6	18
	IQR	3	1	3	1	2	3	1	8.25
Total (n=338)	Median	3	3	4	1	1	1	6	19
	IQR	3	1	2	1	2	3	1	8

The results of the correlation analysis for age and years of education are shown in Table 3. we found that age was a significant negative predictor of the total MoCA score and its subtests. In contrast, we found that years of education were a significant positive predictor of the total score of MoCA and its subtests. Age and years of education explain 12.17 and 24.67% of the total scores of MoCA, respectively.

**Table 3:** Correlation analysis between the Montreal Cognitive Assessment (MoCA) and the demographic variables of age and years of education.

	Age		Years of Education	
	τ_b_	r_2_	τ_b_	r_2_
Visospatial/Executive	-0.22*	0.09*	0.34*	0.16*
Identiﬁcation	-0.17*	0.04*	0.19*	0.06*
Attention	-0.15*	0.04*	0.27*	0.12*
Language	-0.16*	0.04*	0.34*	0.17*
Abstraction	-0.19*	0.06*	0.31*	0.13*
Delayed recall	-0.17*	0.05*	0.18*	0.05*
Orientation	-0.20*	0.05*	0.27*	0.11*
MoCA Total	-0.24*	0.12*	0.37*	0.24*

Note:

* p<0.05.

We found a significant main effect of the age group on the total MoCA scores (H=40, *p*<0.001). Post hoc pairwise comparisons revealed significant differences between the 80+ (Me=15, interquartile range — IQR=7) group and the 60–64 (Me=22, IQR=6.5) (*p*<0.001), 65–69 (Me=21, IQR=8.75) (*p*<0.001) and 70–74 (Me=19, IQR=6) (*p*=0.004) groups. Similarly, significant differences were found between the 60–64 and the 75–79 (Me=19, IQR=10) groups (*p*=0.031).

We also found a significant main effect of the educational level on the total scores of MoCA (H=79.80, *p*<0.001). Post hoc pairwise comparisons revealed significant differences between the primary education group (Me=13, IQR=6) and the secondary (Me=19, IQR=5) (*p*<0.001) and higher education groups (Me=22, IQR=7) (*p*<0.001). Furthermore, significant differences were found between the secondary and higher education groups (*p*<0.001).

### Age and education adjusted norms

Age-adjusted scale scores obtained from our sample are presented in Table 4. Total scores, Pc, *SS*_a_, and scores for the total sample and the five age groups are presented. we suggest cutoff scores to classify cognitive performance following previous standardisations of the test^31^. For the total sample, cognitive performance is considered normal or non-impaired when a raw score above 18 is obtained.

**Table 4:** Scaled scores and percentiles for each age group.

SS_a_	Pc	Suggested cut-off score	Total Montreal Cognitive Assessment Raw Scores					
		Cognitive Impairment	Total sample	60–64	65–69	70–74	75–79	80+
2	<1	Severe	3	-	-	-	-	-
3	1		4–6	-	9	6	3	4
4	2–3		7	10–11	11	7	-	5
5	4–6	Moderate	8–9	13	13	8–10	7	6–7
6	7–12		10–12	14	14	11–12	8	8–10
7	13–20		13–14	15–17	15	14–15	9–11	11–12
8	21–30	Mild	15–16	18–20	16–17	16–17	12–15	13
9	31–43		17–18	21	18–20	18	16–18	14–15
10	44–56		19–20	22	21–22	19–20	19–20	16–17
11	57–68		21–22	23	23–24	21–22	21–22	18
12	60–79		23	24	25	23	23	19–20
13	80–86		24–25	25	26	24	24	21–22
14	87–92	Non impaired	26–27	26	27	26–27	25	23
15	93–95		28	27	28	28	-	26
16	96–97		29	28	29	-	29	27
17	98		-	-	-	-	-	-
18	>99		-	29	-	29	-	28

Abbreviation: Pc, Percentile; SSa, Age-adjusted scaled score.

We found that years of education significantly predicted *SS*_a_ (*r* ^2^=0.239, *p*<0.001) with a coefficient of 0.347. Next^22^, we used this coefficient to calculate *SS*_ae_ for each combination of *SS*_a_ and years of education observed in the sample. we present *SS*_ae_ in Table 5.

**Table 5:** Scaled scores adjusted for age and years of education.

SS_a_	Pc	Years of education																				
		0	1	2	3	4	5	6	7	8	9	10	11	12	13	14	15	16	17	18	19	20
2	<1	5	5	5	4	4	4	3	3	3	2	2	2	1	1	0	0	0	-1	-1	-1	-2
3	1	6	6	6	5	5	5	4	4	4	3	3	3	2	2	1	1	1	0	0	0	-1
4	2–3	7	7	7	6	6	6	5	5	5	4	4	4	3	3	2	2	2	1	1	1	0
5	4–6	8	8	8	7	7	7	6	6	6	5	5	5	4	4	3	3	3	2	2	2	1
6	7–12	9	9	9	8	8	8	7	7	7	6	6	6	5	5	4	4	4	3	3	3	2
7	13–20	10	10	10	9	9	9	8	8	8	7	7	7	6	6	5	5	5	4	4	4	3
8	21–30	11	11	11	10	10	10	9	9	9	8	8	8	7	7	6	6	6	5	5	5	4
9	31–43	12	12	12	11	11	11	10	10	10	9	9	9	8	8	7	7	7	6	6	6	5
10	44–56	13	13	13	12	12	12	11	11	11	10	10	10	9	9	8	8	8	7	7	7	6
11	57–68	14	14	14	13	13	13	12	12	12	11	11	11	10	10	9	9	9	8	8	8	7
12	60–79	15	15	15	14	14	14	13	13	13	12	12	12	11	11	10	10	10	9	9	9	8
13	80–86	16	16	16	15	15	15	14	14	14	13	13	13	12	12	11	11	11	10	10	10	9
14	87–92	17	17	17	16	16	16	15	15	15	14	14	14	13	13	12	12	12	11	11	11	10
15	93–95	18	18	18	17	17	17	16	16	16	15	15	15	14	14	13	13	13	12	12	12	11
16	96–97	19	19	19	18	18	18	17	17	17	16	16	16	15	15	14	14	14	13	13	13	12
17	98	20	20	20	19	19	19	18	18	18	17	17	17	16	16	15	15	15	14	14	14	13
18	>99	21	21	21	20	20	20	19	19	19	18	18	18	17	17	16	16	16	15	15	15	14

Abbreviation: Pc, Percentile; SSa, Age-adjusted scaled score.

Here we present the steps for the interpretation of test scores using our norms:

- locate the individual raw score in the column that matches the individual's age group in Table 4;
- identify the corresponding *SS*_a_ in the leftmost column of Table 4;
- locate the *SS*_a_ in the leftmost column of Table 5;
- identify the *SS*_ae_ in the column that aligns with the individual's years of education in Table 5;
- determine the corresponding Pc and classification for the scaled score obtained in the previous step in Table 4.

For instance, a 65-year-old male participant who achieves a raw score of 17 is assigned a *SS*_a_ value of 8 and a percentile (Pc) range of 21–30, as indicated in Table 4. In this example, if our participant possesses five years of formal education, this would correspond to a *SS*_ae_ value of 10 in Table 5. Consequently, the adjusted score that accounts for both age and education of the participant would be established at 10, which according to Table 4 corresponds to a Pc range of 44–56 and a classification of non-impaired.

## DISCUSSION

The purpose of this study was to standardise the Spanish version of the MoCA for the older population in Lima. Standardising this test in Peru is essential for accurate cognitive evaluation due to the high prevalence of neurodegenerative disorders^1,4^ and the absence of cognitive tests adapted to the sociodemographic and cultural context of the country. Early detection of cognitive impairments like dementia enhances patient prognosis, quality of life, and lessens economic and social burdens^32^, crucial for developing countries.

In line with previous research using MoCA^13,17,22,31^, we found significant effects on overall cognitive performance of age and education, but not sex. Sex differences were found only in identification and attention, with men scoring higher than women, similar to observations in the Chilean population^16^. In addition, we found that education predicts cognitive performance better than age.

Our results support earlier findings that ageing leads to cognitive decline, notably reducing memory, processing speed, and executive functions^33-36^. Additionally, the observed effect of education on cognitive performance supports the view that education and mental engagement may protect against age-related cognitive decline and dementia^37,38^. This highlights concerns about the impact of the pervasive diminished educational attainment and cognitively impoverished environments in Peru^39^.

This seminal study represents the inaugural effort to standardize the MoCA test in Peru, providing a new benchmark for future research on cognitive decline among older adults in the region. The MoCA cutoff score we propose, set at 18, is notably lower than that reported for the Argentinian cohort (25)^17^ and that employed for the Chilean population (22)^16^. This may be due to the higher educational attainment observed in those samples, wherein the majority of older adults attended higher education. In turn, our cutoff is closer to that reported in the Brazilian population (15 for dementia and 19 for mild cognitive impairment)^14^. Overall, our findings are consistent with normative scores adjusted for age and educational attainment observed in studies conducted in Brazil^13^ and Argentina^17^, which report cutoff scores ranging between 24 and 14, contingent upon educational level for this age group. Lower cutoff scores are essential to reduce false positives in low-educated samples, such as ours, as shown by the aforementioned studies.

We observed a notably diminished score on the delayed recall subtest, contrasted by a markedly elevated score on the spatiotemporal orientation subtest. These findings align with prior regional studies indicating that the delayed recall subtest exhibited the most pronounced decline compared to other subtests, while orientation scores demonstrated stability across all age groups^13,16^. Another study revealed that even cognitively deteriorated participants may obtain high scores on the orientation subtest^40^. Moreover, previous studies in the region show that low delayed recall scores are significantly associated with low levels of education^13,14,16^, which are characteristic of our sample. In general, low delayed recall scores are associated with amnestic MCI^41^ and may have a detrimental impact on the daily activities of the evaluated individuals. Future studies should explore the significance and social impacts of this finding in Peruvian older adults.

To our knowledge, this study is the first post-pandemic standardisation of the test in Latin America. Empirical evidence indicates that COVID-19 causes gray matter reduction and a decrease in overall brain size, presumably leading to cognitive alterations^42^. Moreover, there is evidence of social distancing protocols negatively impacting information processing and executive functions^43^. These known effects of the pandemic may partially explain the remarkably low scores observed in our sample compared to previous MoCA standardizations in the region^16,17^. Overall, our results suggest the necessity to update cognitive assessment standards to align with post-pandemic population characteristics for accurate evaluations.

The limitations inherent to our study design must be carefully considered. we stress the limitations of using normative studies lacking diagnostic accuracy analysis for the early identification of patients at risk of developing dementia^19^. For instance, cutoff values of the MoCA derived from normative data have shown very low sensitivity (increasing the rate of false negatives) but high specificity in the assessment of MCI and early dementia, even after adjusting for age and education^44^. we encourage future studies involving Peruvian participants clinically diagnosed with a variety of clinical conditions in addition to healthy controls to determine the optimal cutoff score of the MoCA for different diseases.

In addition, we acknowledge that during our data collection process we did not include standard cognitive evaluation measurements (such as the MMSE or the Functional Activities Questionnaire) to ensure that no participants had significant cognitive deficits in our normative sample. This is expected to reduce the sensitivity of the cutoffs scores derived from our norms. Our efforts to recruit a representative sample of Metropolitan Lima may still under-represent certain demographics, impacting external validity and limiting its use in the wider Peruvian context. Despite its limitations, this study is a crucial step in enhancing cognitive assessment for older Peruvians and highlights the need for further research to address clinical cognitive assessment in Latin America.
