# Erratum: Normative data of the Spanish version of the Montreal Cognitive Assessment (MoCA) in older individuals from Peru

**DOI:** 10.1590/1980-5764-DN-2024-0261-ER

**Published:** 2025-12-01

In the article "Normative data of the Spanish version of the Montreal Cognitive Assessment (MoCA) in older individuals from Peru", DOI https://doi.org/10.1590/1980-5764-DN-2024-0261, published in the Dement Neuropsychol 2025;19:e20240261, page 5:

**Where it was written:**

**Table 4:** Scaled scores and percentiles for each age group.

		Suggested cut-off score	Total Montreal Cognitive Assessment Raw Scores					
SS_a_	Pc	Cognitive Impairment	Total sample	60-64	65-69	70-74	75-79	80+
2	<1	Severe	3	-	-	-	-	-
3	1		4 - 6	-	9	6	3	4
4	2-3		7	10 - 11	11	7	-	5
5	4-6	Moderate	8-9	13	13	8 - 10	7	6 - 7
6	7-12		10 - 12	14	14	11 - 12	8	8 - 10
7	13-20		13 - 14	15 - 17	15	14 - 15	9 - 11	11 - 12
8	21-30	Mild	15 - 16	18 - 20	16 - 17	16 - 17	12 - 15	13
9	31-43		17 - 18	21	18 - 20	18	16 - 18	14 - 15
10	44-56		19 - 20	22	21 - 22	19 - 20	19 - 20	16 - 17
11	57-68		21 - 22	23	23 - 24	21 - 22	21 - 22	18
12	60-79		23	24	25	23	23	19 - 20
13	80-86		24 - 25	25	26	24	24	21 - 22
14	87-92	Non impaired	26 - 27	26	27	26 - 27	25	23
15	93-95		28	27	28	28	-	26
16	96-97		29	28	29	-	29	27
17	98		-	-	-	-	-	-
18	>99		-	29	-	29	-	28

Abbreviation: Pc, Percentile; SSa, Age-adjusted scaled score.

**It should be:**

**Table 4:** Scaled scores and percentiles for each age group.

		Suggested cut-off score	Total Montreal Cognitive Assessment Raw Scores					
SS_a_	Pc	Cognitive Impairment	Total sample	60-64	65-69	70-74	75-79	80+
2	<1	Severe	3	-	-	-	-	-
3	1		4 - 6	-	9	6	3	4
4	2-3	Moderate	7	10 - 11	11	7	-	5
5	4-6		8-9	13	13	8 - 10	7	6 - 7
6	7-12		10 - 12	14	14	11 - 12	8	8 - 10
7	13-20	Mild	13 - 14	15 - 17	15	14 - 15	9 - 11	11 - 12
8	21-30		15 - 16	18 - 20	16 - 17	16 - 17	12 - 15	13
9	31-43		17 - 18	21	18 - 20	18	16 - 18	14 - 15
10	44-56	Non impaired	19 - 20	22	21 - 22	19 - 20	19 - 20	16 - 17
11	57-68		21 - 22	23	23 - 24	21 - 22	21 - 22	18
12	60-79		23	24	25	23	23	19 - 20
13	80-86		24 - 25	25	26	24	24	21 - 22
14	87-92		26 - 27	26	27	26 - 27	25	23
15	93-95		28	27	28	28	-	26
16	96-97		29	28	29	-	29	27
17	98		-	-	-	-	-	-
18	>99		-	29	-	29	-	28

Abbreviation: Pc, Percentile; SSa, Age-adjusted scaled score.

Editor-in-Chief: Ricardo Nitrini http://orcid.org/0000-0002-5721-1525

